# Surveying practicing firearm examiners

**DOI:** 10.1016/j.fsisyn.2022.100228

**Published:** 2022-04-20

**Authors:** Nicholas Scurich, Brandon L. Garrett, Robert M. Thompson

**Affiliations:** a University of California, Irvine, USA; b Duke University, School of Law, USA; c National Institute of Standards and Technology, USA

**Keywords:** Forensic science, Firearms and toolmarks examination, Expert testimony, Error rates, Forensic conclusions

## Abstract

A sample (n = 79) of practicing firearm and toolmark examiners was queried about casework as well as their views about the potential role that statistics might play in future firearm examinations and expert witness testimony. Principal findings include: The modal response for time spent conducting bullet examinations is 2–4 hours, and the modal response for cartridge casings is 1–2 hours. The average participant (median) makes an identification in 65% of casework, makes an elimination in 12% of casework, and reports that the examination was inconclusive in 20% of casework calls. The vast majority of examiners work at laboratories that permit eliminations when class characteristics agree. The reported industry-wide false positive error rate is 1%, though very few participants could name a study or give a citation for their reported estimate. Qualitative responses about the potential role of statistics were mixed.

## 1 Introduction

For over a hundred years, firearm and toolmark examiners have testified in criminal cases in the United States, opining on whether marks imparted on bullets or shell cases suggest that they were fired by a particular firearm [1]. The Association of Firearms and Toolmark Examiners (AFTE), the leading professional association in the field, has promulgated a “theory of identification,” which sets out guidance on how examiners should assess the sufficiency of evidence involved in such work [2]. AFTE also publishes a manual for firearms and toolmark examination procedures [3].

In the past two decades, however, scientific organizations have raised concerns about whether such visual examination methods constitute a foundationally valid discipline, including in two National Academy of Sciences Reports [4,5] and one report by the President's Council of Advisers on Science and Technology [6]. The PCAST Report highlighted one important advance since the 2009 NRC report – a single unpublished study that had been conducted to test the accuracy of judgments made by firearm examiners comparing cartridge cases. The false positive error rate observed in that study was estimated as 1 in 66, with a confidence bound as high as 1 in 46, and a much higher inconclusive rate: over 1 in 3 (Table 2, p. 111). The PCAST report concluded that the method was not sufficiently validated on the basis of this single study. In response to these types of concerns, judicial rulings, and new U.S. Department of Justice Guidelines, firearm examiners in some jurisdictions have tempered the description of their conclusions in criminal court testimony [[7], [8]].

There has recently been much discussion and debate about firearm examiner error rate studies and how to interpret the findings (e.g., Ref. [9]. Claims are sometimes made that error rate studies differ from actual casework in material ways, including the quality of the evidence samples, the types of comparisons that are conducted, and the relative frequency with which certain conclusions (e.g., inconclusive conclusions) are reached. Importantly, these claims are made without any empirical support. For example, a document released by the Department of Justice [10] advocates for a particular type of study design by repeatedly asserting that the design “simulates real casework” and “replicate[s] casework conditions,” but the document does not provide a single citation to support the claim regarding what “real casework” entails (see paragraph 2, p. 20). In fact, there are no systematic studies of firearm examiner casework. Very little is known empirically about how such experts view their casework, the variety in the type of evidence that they examine, the range of conclusions that they typically reach in their casework, and what practical challenges arise in their work. The present study seeks to address this knowledge gap by surveying a group of practicing firearm and toolmark examiners to better understand the conditions surrounding their casework.

## 2 Literature review

Several surveys have been conducted to examine how other forensic professionals view their work. For example, Murrie et al. [11] queried 183 forensic examiners about error rates in various forensic disciplines; the participants included examiners from a range of disciplines, including biology (84), pattern evidence (43), chemistry (32), and crime scene investigation (12). The results varied widely from some examiners reporting that a false positive error “is impossible” to some reporting that false positive errors occur 50% (1 in 2) of the time (See Fig. 1, p. 5) for pattern evidence. Notably, when asked to provide a source for their error rate estimate, 79% of the examiners were not able to cite a source, and only 12 examiners could point to a specific journal article or study. Overall, the participants estimated that false positive errors were less frequent than false negative errors in their respective disciplines because their disciplines placed more weight on seeking to minimize false positive errors.

A survey by Wilkinson and Swinnett [12] probed forensic hair examiners’ opinions about their discipline and casework. Fifty-eight examiners from nine different countries completed the survey. The results revealed that, despite considerable criticism of the validity of hair comparison analysis, only two of the participants reported that they no longer receive hair evidence as part of their casework. The vast majority of participants (90%) reported that they undergo proficiency testing, and the results were mixed as to what framework of guidance they use to conduct hair comparisons (e.g., the Forensic Human Hair Guidelines by the Scientific Working Group for Materials Analysis (SWGMAT) or the European Network of Forensic Science Institutions (ENFSI) Best Practice Manual for the Microscopic Examination and Comparison of Human and Animal Hair). No participant in the study used a statistical approach or attempted to quantify their results, asserting that “numbers cannot be applied to microscopic features” (1 participant), “characteristics are a form of continuous variation” (1 participant) and that there are “too many variables to consider” (1 participant) (p. 12).”

Other research work has examined archival data concerning forensic examiners' casework products to gauge practices. A study of two years’ worth of data from case processing of fingerprint examiners at the Houston Forensic Science Center crime lab generated descriptive data regarding the typical number of prints examined in a given case and the range of conclusions reached by examiners [13]. In analyzing 5430 prints determined “to be of value,” Rairden et al. [13] found that 60% of latent comparisons resulted in an identification, 28% were exclusions, and the other 12% were inconclusive. Although only 3% of all cases had a “consultation,” the authors reported the results of the consultation as follows:

The modal outcome was an exclusion changed to an identification (n = 22 prints), followed by an exclusion changed to an inconclusive (n = 16 prints). The next three most frequent consultation decisions concerned the threshold between identification and inconclusive, followed by inconclusives changed to exclusions. There were no instances in which an identification was changed to an exclusion. (p. 219)

The number of years of experience or seniority was not associated with whether the initial decision was changed following the consultation.

Bali et al. [14] analyzed a random sample of 500 reports from *Collaborative Testing Services Incorporated* (CTS) proficiency tests in eight forensic disciplines: 43 conclusions in fiber analysis, 121 in firearm examination; 33 in glass analysis; 52 in handwriting examination; 39 in paint analysis; 59 in questioned documents examination; 64 in shoeprint impression evidence; and 89 in toolmark examination. They were specifically interested in how the conclusion was described in the report (e.g., a categorical conclusion or a random match probability). Overall, a strong majority of the conclusions were stated in categorical terms. However, in firearm examination, an overwhelming majority (95.9%) gave a categorical conclusion, and less than half of the reports articulated reasons for reaching that conclusion. Bali et al. [14] do note that reliance on CTS results has several limitations, including the possibility that forensic examiners may have omitted information in a CTS report that is required by laboratory reporting policy (see pp. 222–223).

Cole and Barno [15] examined expert reports and transcripts regarding friction ridge prints (91 reports), firearms and toolmarks (48), questioned documents (52), and shoeprint comparisons (381 reports from CTS), obtained from a variety of sources (e.g., a search of Westlaw for expert materials), in the United States. Of the 48 firearm and toolmark reports, 28 (58%) reported an identification, 14 (29%) reported an inconclusive, and 6 (13%) reported an exclusion. All of the firearm and toolmark examiner reports followed the AFTE range of conclusions (i.e., identification, inconclusive, or exclusion). Cole and Barno [15] concluded that they overall “found relatively few probabilistic reports. The probabilistic reports that we did find were almost entirely 'Inconclusive' reports which, by their very nature, were always coded as probabilistic (p. 412).”

## 3 Present study

Although firearm examinations are one of the most commonly conducted forensic analyses (DOJ, 2014), and there has been an increasing number of error rate studies (e.g. [16]), and academic commentary on the technique [17,18], little is known about the actual practices of firearm examiners in conducting case work, such as the average amount of time spent on case work, whether their lab policies follow the AFTE procedures precisely or have a modified AFTE protocol, the frequency with which examiners testify in court, how often certain conclusions are reached, etc. Our aim was to gain insights into these areas. We were also interested in learning what firearm examiners believe impacts the quality of their work and what issues judges and jurors misunderstand about their profession, as well as the relevance of novel technology and statistical models to firearm examination. In short, the primary objective of this study is to better understand the practices and perceptions of actual firearm examiners.

This study also seeks to understand what firearm examiners perceive to be the industry-wide false positive error rate for firearm examination – an issue that judges consider when assessing the admissibility of expert testimony under the *Daubert* standard. These values might also be articulated to the jury during testimony. The study by Murrie et al. [11] included 43 “pattern evidence” examiners, but it did not separate the responses of experts by their domain (i.e., it combined latent print examiners, document examiners, and firearm and toolmark examiners), so it remains unknown as to what firearm and toolmark examiners think about the false positive error rate and the bases for those estimates.

## 4 Methods

### 4.1 Participants and procedure

The survey was posted on the online AFTE Member Forum from July 2020 until November 16, 2020. Participation was anonymous, voluntary, and uncompensated. There were 129 survey initiations. Participants who spent less than 3 min on the survey (n = 34) or spent more than 3 h (n = 5) or who did not respond to any questions (n = 11) were excluded from all analyses reported hereinafter. A total of 79 participants satisfied these criteria and were included in the analyses reported below. The mean time to complete the survey was 19.6 min.

The average participant (median) had been an AFTE member (no matter the status) for 12 years (with a range of 2–32 years) and had attended 5 AFTE training conferences (with a range of 0–23). To gauge experience, we also asked, how many years had they conducted unsupervised firearms and/or toolmark examinations after they were trained, and they reported a median of 12 years (with a range of 0–32 years). The average participant reported working on 90 cases per year as the primary examiner (mean = 76, SD = 29.5), and testifying in court on average 5 times per year (with a range of 0–20). One third of the participants stated that they are currently certified by AFTE in firearm examination. AFTE certification is different than simple membership in that it requires among other things the passing of several additional examinations, completion of a training curriculum, casework experience, and a bachelor's degree (see https://afte.org/afte-certification/certification-program). Certified AFTE members have been described as “journeymen-level” examiners.

To learn more about their workplaces, we asked, if they currently work at a crime lab, how many full-time employees work at the lab (not limited to just firearm examiners). We received the following responses: 13% of participants worked at a lab with 10 or fewer employees; 18% worked at a lab with 11–20 employees; 41% worked at a lab with 21–50 employees; and 25% of participants worked at a lab with more than 50 employees (3% of participants selected “NA”). According to the last Bureau of Justice Survey over two-thirds of labs had fewer than 24 employees and 78 of 404 labs (19%) had 50 or more employees [19], the participants in the present study tended to work in relatively large labs.

We asked, “In your case work, are you required to follow the AFTE examination procedure manual or does your lab have its own examination procedures for firearms examinations?” [3]. Twenty percent of participants work in a lab that follows the AFTE procedures, while 80% work in a lab that follows a modified-AFTE procedure manual. We also asked, “As a policy, does your lab permit you to declare an exclusion or elimination when class characteristics are similar?” Most participants (87.3%) replied yes, 11.4% replied no, and 1.3% (n = 1) replied “I don't know.” Three participants reported that their lab follows the AFTE procedure manual and yet they are not permitted to report an exclusion or elimination when class characteristics are similar.

## 5 Results

### 5.1 Casework characteristics

Recall that the average participant works as the primary examiner on 90 cases per year. Fig. 1a below displays how much time participants reported working on a typical firearms case examining bullets and Fig. 1b displays how much time they reported working on a typical case examining cartridge cases. Note that the response options are the five categories listed on the x-axis.

**Fig. 1a:**
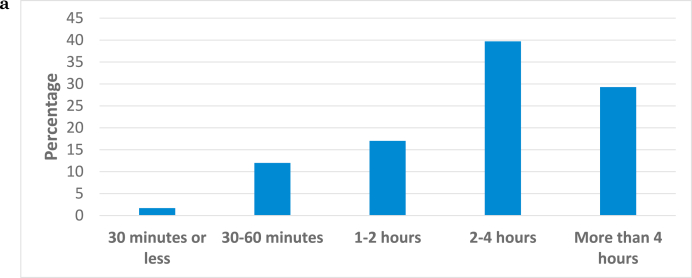
How much time do you spend on a typical firearms case examining bullets?.

**Fig. 1b:**
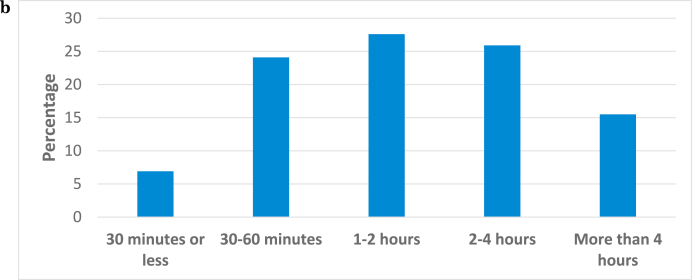
How much time do you spend on a typical firearms case examining cartridge cases?.

Although there is variability in the responses, the modal response for bullet examinations is 2–4 hours and the modal response for cartridge casings is 1–2 hours. The difference between the time spent on comparing bullets versus comparing cartridge cases is statistically significant (Kruskal's gamma = 0.872, p < .001).

We also asked, “How often does a verifier disagree with a conclusion you reach in your casework?” There were six possible response options: always; most of the time; about half of the time; sometimes; rarely; never. The majority of participants (72.4%) indicated “rarely” and 22.4% indicated “never.” No participants selected “about half of the time” or “sometimes” while 1.7% selected “most of the time” and 3.4% selected “always.” These latter two categories of responses are difficult to interpret and may reflect a misunderstanding of the question. What is clear, however, is that verifiers rarely if ever disagree with the conclusion reached by firearm examiners. Also, there was no distinction as to what conclusions were necessarily verified by their laboratory SOP; identifications, exclusions, inconclusives, or a mixture of these.

**Fig. 2:**
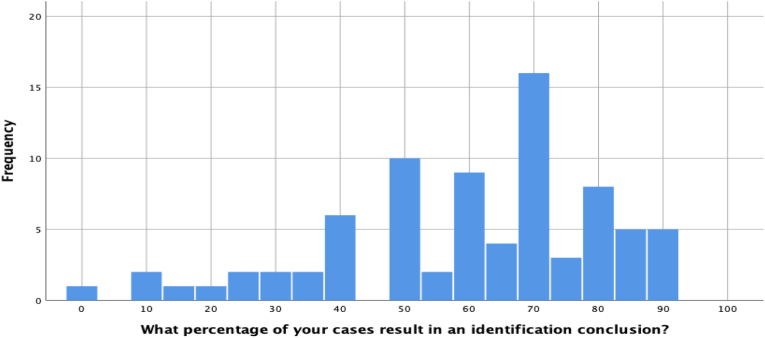
Percentage of identification conclusions reached in casework.

We next examined the percentage of conclusions participants reach in casework. We asked for each type of conclusion (i.e., identification, elimination, inconclusive) separately. The responses for identification conclusions are plotted in Fig. 2 below. The median response was 65%, though considerable variability was observed. One participant selected 0%, which is difficult to interpret and possibly a misunderstanding, while the highest reported value reported by a single participant was 92%. Four participants reported that 90% of their conclusions in case work were identifications.

**Fig. 3:**
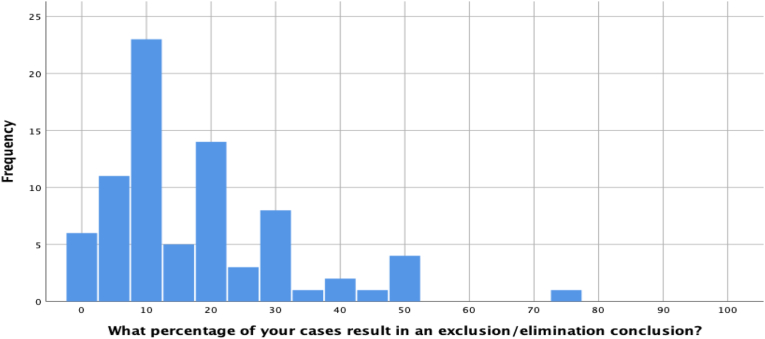
Percentage of elimination conclusions reached in casework.

Far fewer elimination conclusions were reported by participants. The median response was 12%, though, again, considerable variability was observed, which is depicted in Fig. 3 below.

**Fig. 4:**
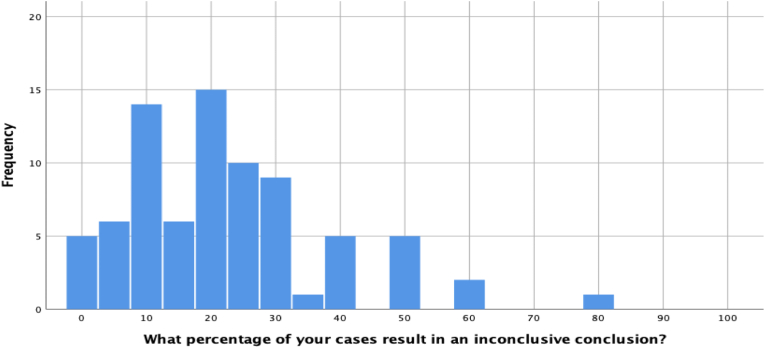
Percentage of inconclusive conclusions reached in casework.

The average participant (median) reported that 20% of their conclusions in casework are inconclusive conclusions. Fig. 4 displays the reporting regarding percent of cases with inconclusive conclusions.

It is perhaps surprising that inconclusive conclusions (median = 20%) are more frequent than elimination conclusions (median = 12%) in casework. This could simply reflect the type of evidence that is submitted to laboratories in casework (e.g., perhaps many severely degraded samples or fragments are submitted to the laboratory for review even though they cannot be compared). We emphasize that these survey results cannot speak to the correctness or incorrectness of the reported conclusions.

However, an alternative explanation of this finding is that inconclusive conclusions are more common than elimination conclusions because some laboratories do not permit firearm examiners to call elimination when class characteristics are in agreement and instead require examiners to call inconclusive. Consistent with this hypothesis, the median number of inconclusive responses for participants whose lab permits them to call elimination when class characteristics agree is 20% whereas the median number of inconclusive responses for examiners who are not permitted to call elimination when class characteristics agree is 40%. Bear in mind that the majority of participants (87.3%) work in labs that do permit examiners to call elimination when class characteristics agree, which is consistent with the AFTE protocol. Additionally, examiners know that the ammunition-firearm pairing can have a pronounced influence in the quality and reproducibility of firearm toolmarks imparted on firearm evidence. The term “ammunition-firearm pairing” is used here to describe a particular firearm and a particular cartridge combination in a firing event. Most firearms can fire cartridges of many manufacturers, brands, loadings, and materials. These variables will often affect the toolmarks reproduced on fired bullets and cases. It is also not uncommon that when a suspect firearm is test fired, the resulting bullets or cartridge cases still cannot be identified when microscopically compared. This is one scenario in casework that describes the challenge in determining categorical exclusion results in casework, especially when comparing fired bullet evidence.

### 5.2 Perceptions of false positive error rates

We next asked questions about error rate studies regarding firearms and toolmark work in general. First, we asked, “What would you estimate the industry-wide false positive (false identification) error rate is for firearm analysis?” Participants were instructed to enter their response as a percentage into an open text box. Approximately one third (36%) of participants did not respond to this question. Of those participants who did respond, the median (and modal) response was 1% with a range of 0.01%–10%. The responses did not vary based on whether an individual was AFTE certified (mean = 1.75, standard deviation (sd) = 2.36) or not (mean = 1.55, sd = 1.49), t = −0.376, df = 48, p = .708.

Related to the error rates reported above, we asked, “Have you read any of the error rate studies regarding firearm/toolmark examination done by human firearm examiners using microscopy (i.e., not using 3D digital imaging technology)? Can you list a few that you have read?” Thirty percent of participants (n = 24) did not respond to this question, while six participants (7.5%) stated “no” and 19 participants (24%) stated “yes” but did not list any studies. Of the participants who did list studies, they tended to list several studies, though often they gave very incomplete information (e.g., sometimes just saying “studies by Hamby”).

Despite the incomplete and sometimes indecipherable information, we attempted to count the number of times certain studies were referenced, and we stress that these numbers are approximations due to uncertainty in spelling, missing information, etc. Nonetheless, the Baldwin et al. [20] study was referenced 17 times, “Hamby”/”Brundage” [[21], [22]] was referenced 15 times, “Fadul” (or the “Miami study”) [23] was referenced 5 times, the Smith, Smith and Snipes [24] study was referenced 4 times, and the Keisler et al. [25] study was referenced 2 times. Bear in mind that these responses were provided by 30 participants, which is approximately 38% of the total sample.

### 5.3 Qualitative responses: Factors affecting casework

Participants were asked “Please list and/or describe any factors that tend to impact the quality and quantity of your work (e.g., time pressure/backlogs/court dates; the quality of the evidence submitted; quality of available equipment, etc.).” Representative responses are provided below (note: grammatical and typographical errors appear in original):

• The quality of the evidence submitted.
• The quality of available equipment
• We have increasing pressure to speed up our turnaround time, with a mounting backlog. We have dated microscopes that are constantly on the fritz and lending to less than desirable photos.
• Nothing impacts the quality of my work. My work ethic will not allow any external factor to effect the quality of my work. Quantity on the other hand can be effected by several factors: quality of evidence submitted, volume of evidence submitted, volume of unnecessary administrative paperwork/regulations created by agency as well as accrediting body.
• Whether bullets are washed after autopsy or not, crime scene response/reports, other lab/training requirements
• Sometimes the evidence recovered has been exposed to the elements for a long time (and may not even actually be associated with the case being worked) and have been run over by vehicles/lots of damage.

Overall, these responses highlight that laboratory examiners do not work in a vacuum and often depend on separate non-laboratory police staff to collect evidence from crime scenes. That work can very seriously challenge the lab examiners. However, turning to the work of the lab examiners, they did not emphasize factors that are more within their control.

### 5.4 Qualitative responses: What do jurors and judges misunderstand about firearm analysis

We asked: What are the primary issues that you feel lawyers, judges, or jurors misunderstand about firearm analysis? Representative responses included:

• That the probability of a bullet or cartridge case truly being identified to two or more firearms is zero
• Subclass does not always prevent an examiner from making a proper identification even if it is present. "Subjective" and "opinion" are not the same as guessing.
• That our opinions are based on years of empirical studies
• They don't understand how much knowledge we have to have about the manufacturing process of the firearm, and everything that leads to the production of the marks we look at.
• They are misleading about the science and overstep their bounds on something they are not educated or trained in. A judge would not argue that a doctor was wrong in his analysis, but would look to see if another doctor(s) would have made the same call to determine if that doctor is credible.
• That you cannot apply techniques from DNA to Firearms Identification.
• Pretty much all of it. Especially, when people from CSAFE, PCAST, and the NAS don't take the time to understand it fully, and decide that the PhD behind their name qualifies them to render an opinion in court on it.

These responses suggest a range of reactions regarding misperceptions and communication with lawyers and jurors. Some respondents feared that their work is perceived as stronger and more probative than is warranted, e.g., that there is a “zero” probability of an error. Others, however, felt that their knowledge and skill is under-appreciated, not over-valued, and expressed a defensive concern that people with PhDs in other disciplines have themselves raised concerns about their work.

### 5.5 Qualitative responses: What role might statistics play in firearm analysis

Finally, we asked, “Do you have any thoughts on the role that statistics could play in firearms and/or toolmarks work in the future?” Representative responses included:

• They could likely be helpful (see what I did there?). But, if presented to a jury, all bets are off. Most people have little background in stats and would find it more confusing. They just want to know, "does the bullet match the gun" in my opinion. Thats the information they need in order to make an informed decision.
• Statisticians would need to agree on a method. I think it is difficult to determine a way that firearms identification can be analyzed statistically.
• It would be nice to give a confidence number and a cutoff for inconclusive or exclusions
• Developing some type of probability scores based off of 3D topography would be very useful in supporting source conclusions and giving the legal system a way to interpret what an “identification” means beyond the AFTE definition that “the likelihood that another firearm fired the evidence is so remote as to be considered a practical impossibility”.
• I think that it shouldn't play a role in the field ever. Firearms/toolmarks identification is not like DNA or Chemistry and we should not be trying to make it that way. The field is unique in it's ability and we should focus on strengthening our responses to criticism versus attempting to appease the oppositions.
• 3D systems and the data they analyze would need to improve drastically to have any useable information; NIST projects seem to produce interesting results, but the datasets are too small; the largest database of images is in NIBIN, but the IBIS algorithms are so inconsistent that I'd never trust any analysis from that data--specifically their abitly to determine similar from different. Any user has horror stories about how bad thier systems can be at times in missing true matches.
• I don't think it should play a role. Firearms identification has been just fine for decades without assigning empty values to an identification.
• Statistics can play a role in our field, but i do not believe that they are the end all be all as many people think. When comparing toolmarks we are dealing with the random creation of imperfections transferred from one object to another. Therefore, any statistics that are applied will have to be either theoretical probabilities or some form of similarity score generated from 3D topographies. Where the 3D similarity scores will falter is who will define what is similar enough as well as the computers ability to deal with damaged samples.
• I think they would be great to have. Especially given the current climate around forensics. I think it might be beneficial for statistician to go through the complete training program for firearms and tools marks and then try to apply statistics to what they have learned, instead of it being the other way around where a stats person tries to apply stats to something they don't understand.

## 6 Discussion

In the United States, crime laboratories perform large numbers of firearm comparisons each year; in the most recent Bureau of Justice data from 2014, there were over 142,000 firearm and toolmark examinations completed [26]. For local or municipal labs, 10% of their requests were for firearms work (Id.). Given the prominence of firearms-related work, including in criminal cases involving gun violence, empirical work in understanding firearm examinations has been long overdue. This survey is the first of its kind, designed to gather information directly from practicing firearms examiners. As described, we focused on the leading professional association of firearms examiners, AFTE, and we obtained participation from professionals who tended to have an experience in larger labs.

We begin by noting that this survey has a number of limitations. Participation was voluntary, self-selected, and solicited across AFTE, and while participants had extensive representative experience, we do not know, due to anonymity, whether they worked at a representative range of crime laboratories. Most reported working at larger crime laboratories. Further, we asked for their self-reported assessments of their work; as a result, they may not accurately estimate or recall certain information, for example the number of hours they work on typical cases, or how many cases they work on in a given year. The strength of this method is that we were able to obtain candid reflections on the field from a number of active practitioners. The open responses in particular display a willingness to openly engage in discussing some of the most pressing issues in the discipline. We also did not specifically ask if the participants work in laboratories in the United States.

The results of the current study provide important insights for both academics studying the firearm examination discipline as well as for firearm examiners. For example, participants consistently reported as fact that there is a 1% industry-wide false positive error rate. Yet many participants were not able to name a specific study to support this observation. As noted, judges in *Daubert* jurisdictions might consider the error rate in determining the admissibility of firearm examiner testimony, and it would be troubling for an expert witness to express an error rate but be unable to explain the basis for that opinion with reference to a scientific study. The findings suggest that examiners might benefit from reading and understanding results of error rate studies. That said, a large number of participants declined to answer this question in the survey, which itself may signal discomfort with the issue. However, quite a few respondents were familiar with specific studies regarding accuracy of the firearms discipline.

One of the most hotly-debated issues in the firearm and toolmark examination field concerns the inconclusive responses in extant error rate studies [9,27,28]. Two studies have found 20% [25] to 33.7% [20] of different-source comparisons resulted in inconclusive responses. The present study found that the average participant reported that 20% of calls in casework are inconclusive, and the percentage doubled among participants who work in a laboratory that does permit examiners to call elimination when class characteristics agree. Unlike the error rate studies where ground-truth is known and there are two ground-truth categories of evidence (same source or different source) by design, ground truth is not known in case work and thus one cannot know whether the percentages reported in this study reflect accurate inconclusive calls, nor can one know whether the inconclusive responses in case work are predominantly in response to different-source comparisons, as they are in the extant error rate studies. Nevertheless, given the frequency with which it appears in casework, and the ambiguous interpretative issue in controlled studies, inconclusive conclusions most definitely merit further discussion and study.

Lessons can be learned from these data for both academics and practitioners. For practitioners, it is of paramount importance that the jury be apprised of laboratory policies regarding eliminations and inconclusive conclusions, and specifically if eliminations are not permitted in the presence of class characteristics that are in agreement, the jury must be informed that an “inconclusive” is being reported but this is because the laboratory policy does not permit the call of elimination. It is also appropriate to explain to the jury this and any other deviation from AFTE guidelines.

For academics and researchers, the findings in this survey coupled with the extant error rate studies suggest a need to study inconclusive responses specifically as they amount to significant percentage of decisions in casework. There is also some evidence to suggest that examiners are not able to apply the AFTE concept of inconclusive reliably. AFTE has three different categories of inconclusive responses (see https://afte.org/about-us/what-is-afte/afte-range-of-conclusions):

Inconclusive-A: Agreement of all discernible class characteristics and some agreement of individual characteristics, but insufficient for an identification.

Inconclusive-B: Agreement of all discernible class characteristics without agreement or disagreement of individual characteristics due to an absence, insufficiency, or lack of reproducibility.

Inconclusive-C: Agreement of all discernible class characteristics and disagreement of individual characteristics, but insufficient for an elimination.

First, note that the extant error rate studies have not reported the various types of inconclusive responses. They combined the three different types of inconclusive responses and simply reported how many inconclusive conclusions occurred in the study despite the fact that they asked participants to indicate the type of inconclusive response (see Ref. [20]; pp. 30–33). Second, one recent study using 3-D imagining technology to make comparisons of cartridge cases did actually disaggregate the inconclusive responses by type and found that 12% of the responses for different-source comparisons were Inconclusive A or B whereas 25% of all the responses were Inconclusive C (Chapnick et al. [29], Table 2). Complete reporting of the data in this manner is appropriate and future error rate studies should follow suit. Additionally, it is important to note that the inconclusive responses were selected 37% of the time for different source comparisons, and among those 1/3 of the responses were Inconclusive A or B. These numbers are significantly higher than the 3 identification decisions reached in response to a known different-source comparison, and thus merit more research and evaluation.

The open-ended responses that we received describe a range of views regarding a forensic discipline that is in flux. Some participants tended to view quality control problems as largely outside of the lab and outside of their control. On the other hand, virtually none expressed quality control issues in their own work. However, others expressed concern that lawyers and jurors assume that their work is more accurate and foolproof than it really is. In contrast, some expressed a defensiveness about their discipline, and a concern with other research scientists with PhDs levelling critiques. Some participants described great willingness to incorporate new statistical or three-dimensional imaging techniques into their process and their conclusions. Others expressed real skepticism whether lawyers and jurors could understand such statistical approaches or whether they could be developed.

We note that these problems of communications may run in both directions: in a recent survey of judges, judges themselves expressed strong interest in further forensic science education and also real needs in terms of ability to access material concerning the reliability of forensic science methods [30]. In our survey, a number of participants strongly preferred the status quo, opining that the discipline has successfully performed for decades without a need for change. These results suggest a wide variety of thinking and opinion among practitioners on some of the most pressing topics in forensic evidence today.

## 7 Conclusion

This first-time survey of firearms practitioners provides insight into the work, the practices, and the culture within an important forensic discipline. These results suggest that it would be highly valuable to obtain more case processing information about the actual casework of firearms examiners to better understand the range of work done and conclusions reached in practice. These results suggest that lab policies may affect the conclusions reached: they structure and may guide the ultimate results reported by examiners. Further work should explore the impacts of lab policies on examiners. Finally, the open-ended responses suggest a culture shift in the discipline with a broad mix of responses regarding the introduction of statistical and technological approaches.

## Funding

This work was partially supported by the Center for Statistics and Applications to Forensic Evidence (CSAFE) through Cooperative Agreement #70NANB15H176 between the 10.13039/100000161National Institute of Standards and Technology and 10.13039/100009227Iowa State University, which includes activities carried out at 10.13039/100008047Carnegie Mellon University, 10.13039/100006510Duke University, 10.13039/100008476University of California, Irvine, and 10.13039/100008457University of Virginia. We thank Robert Ramotoski, Katherine Sharpless, Xiaoyn (Alan) Zheng, John P Jones at NIST for helpful comments on a draft of this manuscript. We also wish to thank Nancy McCombs, Past AFTE President (2019-2020), for helping facilitate data collection.

## Declaration of competing interest

The authors declare that they have no known competing financial interests or personal relationships that could have appeared to influence the work reported in this paper.
